# Asialoglycoprotein receptor 1 promotes SARS-CoV-2 infection of human normal hepatocytes

**DOI:** 10.1038/s41392-024-01754-y

**Published:** 2024-02-14

**Authors:** Xinyi Yang, Xu Zheng, Yuqi Zhu, Xiaying Zhao, Jun Liu, Jiangna Xun, Songhua Yuan, Jun Chen, Hanyu Pan, Jinlong Yang, Jing Wang, Zhimin Liang, Xiaoting Shen, Yue Liang, Qinru Lin, Huitong Liang, Min Li, Fei Peng, Daru Lu, Jianqing Xu, Hongzhou Lu, Shibo Jiang, Ping Zhao, Huanzhang Zhu

**Affiliations:** 1 State Key Laboratory of Genetic Engineering and Engineering Research Center of Gene Technology, Ministry of Education, Institute of Genetics, School of Life Sciences, Yiwu Research Institute, Fudan University, Shanghai, China; 2 Department of Microbiology, Faculty of Naval Medicine, Naval Medical University, Shanghai, 200433 China; 3 Scientific Research Center, Shanghai Public Health Clinical Center, Fudan University, Shanghai, China; 4 Department of Infectious Diseases and Immunology, Shanghai Public Health Clinical Center, Fudan University, Shanghai, China; 5 Changzheng Hospital, Naval Medical University, Shanghai, China; 6 Key Laboratory of Medical Molecular Virology (MOE/NHC/CAMS), Shanghai Institute of Infectious Disease and Biosecurity, School of Basic Medical Sciences, Fudan University, Shanghai, China; 7 Department of Infectious Diseases and Nursing Research Institution, National Clinical Research Center for Infectious Diseases, The Third People’s Hospital of Shenzhen, Shenzhen, Guangdong China

**Keywords:** Infectious diseases, Infectious diseases

## Abstract

Severe acute respiratory syndrome coronavirus 2 (SARS-CoV-2) causes multi-organ damage, which includes hepatic dysfunction, as observed in over 50% of COVID-19 patients. Angiotensin I converting enzyme (peptidyl-dipeptidase A) 2 (ACE2) is the primary receptor for SARS-CoV-2 entry into host cells, and studies have shown the presence of intracellular virus particles in human hepatocytes that express ACE2, but at extremely low levels. Consequently, we asked if hepatocytes might express receptors other than ACE2 capable of promoting the entry of SARS-CoV-2 into cells. To address this question, we performed a genome-wide CRISPR-Cas9 activation library screening and found that Asialoglycoprotein receptor 1 (ASGR1) promoted SARS-CoV-2 pseudovirus infection of HeLa cells. In Huh-7 cells, simultaneous knockout of *ACE2* and *ASGR1* prevented SARS-CoV-2 pseudovirus infection. In the immortalized THLE-2 hepatocyte cell line and primary hepatic parenchymal cells, both of which barely expressed ACE2, SARS-CoV-2 pseudovirus could successfully establish an infection. However, after treatment with ASGR1 antibody or siRNA targeting ASGR1, the infection rate significantly dropped, suggesting that SARS-CoV-2 pseudovirus infects hepatic parenchymal cells mainly through an ASGR1-dependent mechanism. We confirmed that ASGR1 could interact with Spike protein, which depends on receptor binding domain (RBD) and N-terminal domain (NTD). Finally, we also used Immunohistochemistry and electron microscopy to verify that SARS-CoV-2 could infect primary hepatic parenchymal cells. After inhibiting ASGR1 in primary hepatic parenchymal cells by siRNA, the infection efficiency of the live virus decreased significantly. Collectively, these findings indicate that ASGR1 is a candidate receptor for SARS-CoV-2 that promotes infection of hepatic parenchymal cells.

## Introduction

Coronavirus disease 2019 (COVID-19), an acute infectious disease caused by SARS-CoV-2, has created a global pandemic and public health crisis since December 2019.^1^ SARS-CoV-2 preferentially infects cells of the respiratory tract,^2^ but SARS-CoV-2 could also be detected in multiple organs, including the liver, brain, heart, kidney, and intestine.^3–9^ Alongside respiratory symptoms, SARS-CoV-2 causes multi-organ dysfunction that includes hepatic dysfunction, which has been observed in over 50% of COVID-19 patients.^10–13^ Liver injury results from the influx of cytokines, including IL-6, caused by the patient’s systemic response, or by direct viral infection of the liver.^7,10^ Using Droplet Digital PCR (ddPCR) at autopsy, Stein SR et al. found that SARS-CoV-2 could infect multiple organs, including the liver,^9^ and Wang et al. found the presence of intracellular virus particles within hepatocytes in the autopsies of COVID-19 patients through electron microscopy,^7^ suggesting direct cytopathy of SARS-CoV-2 in hepatocytes.

Similar to SARS-CoV, SARS-CoV-2 enters lung cells mainly through interactions between viral Spike protein and cell-surface ACE2 receptor.^14,15^ Through in vitro experiments, multiple liver-derived cell lines could be infected with SARS-CoV-2, but some studies showed that hepatocytes barely express ACE2.^16–18^ These results indicate that other important host receptors might exist that bind to different domain(s) of SARS-CoV-2 Spike and promote the entry of SARS-CoV-2 into hepatocytes.

In previous studies, researchers also discovered some potential SARS-CoV-2 receptors,^19,20^ such as AXL,^21^ NRP1,^22^ ASGR1,^23^ CD147,^24^ KIM-1,^25^ TRF^26^ and the latest report TMEM106B.^27^ Except for ASGR1, the expression levels of other reported potential receptors in hepatocytes are low.^18^ However, ASGR1 is highly expressed in hepatocytes, there is still a lack of direct evidence of SARS-CoV-2 infecting hepatocytes by ASGR1 or other receptors.

CRISPR-based genome-wide screening technology has been successfully applied to screen multiple receptors involved in viral infection and key host factors.^28–30^ In addition, a genome-wide CRISPR knockout library has been used to screen potential host factors important in SARS-CoV-2 replication.^31–34^ Unlike the CRISPR library based on gene knockout, the CRISPR activation library is a gain-of-function screen that could be used to identify a non-ACE2 receptor associated with SARS-CoV-2 infection in a cell model that does not express ACE2.^35,36^ Therefore, we herein generated genome-wide CRISPR-activated HeLa cell libraries and uesd SARS-CoV-2 pseudovirus to screen new non-ACE2 receptors for SARS-CoV-2. We then blocked the expression of candidate receptors to determine if SARS-CoV-2 pseudovirus infection could be prevented in human hepatocyte cell lines and primary hepatocytes. Importantly, we further verified whether SARS-CoV-2 could infect through candidate receptors in human primary hepatocytes. Taken together, our data indicate that ASGR1 is a novel host functional receptor in hepatocytes for SARS-CoV-2.

## Results

### Genome-wide CRISPRa screening identifies new potential receptors for SARS-CoV-2 pseudovirus

**Fig. 1:**
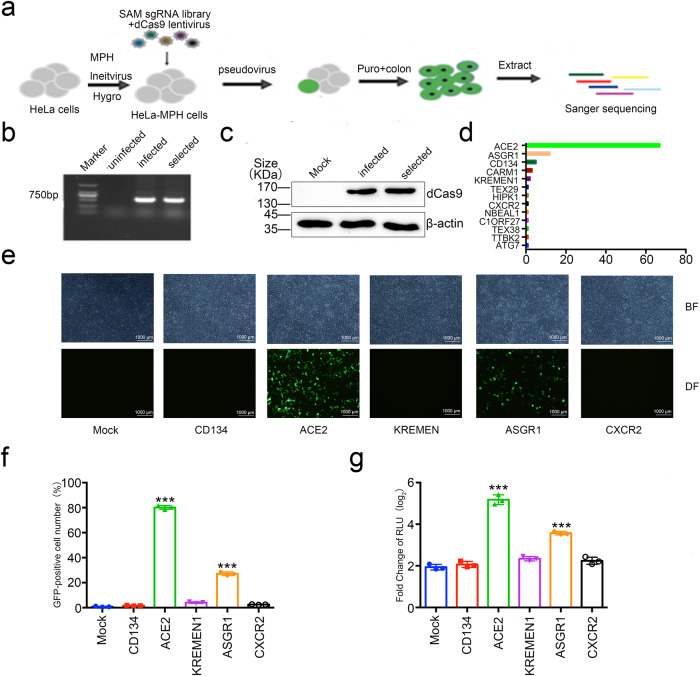
A pooled, genome-wide CRISPR screening for candidate receptors involved in SARS-CoV-2 entry. **a** Outline of genome-wide activation CRISPR screen strategy. HeLa cells were infected with MPH-lentivirus able to express MS2-p65-HSF1 proteins treated with Hygromycin B for fourteen days. Then, HeLa cells expressing MS2-p65-HSF1 were infected with lentiviral dCas9 and sgRNA library targeting 20234 human genes. Fourteen days post-screening with blascicidin, genomic DNA was extracted from monoclonal cells after puromycin selection. Candidate genes were identified by Sanger sequencing. **b** By extracting the genomes of cells in different treatment groups and using specific primers targeting MS2-p65-HSF1 for PCR identification, it was verified that the MS2-p65-HSF1 gene was stably integrated into the infected group cells. **c** After infection with HeLa cells expressing MS2-p65-HSF1 with SAM library, Western blot of cell lysate with anti-Cas9 antibody was performed to identify the expression of dCas9 protein. **d** Statistical analysis of candidate sgRNAs. The genomes of all monoclonal cell lines were extracted, and the corresponding sgRNA sequences were amplified using primers targeting sgRNA sequences and subjected to Sanger sequencing. The candidate genes corresponding to sgRNAs were ranked according to abundance. **e**–**g** ASGR1 facilitates SARS-CoV-2 virus pseudotype with wild-type Spike infection as potently as ACE2. HEK293T cells were transfected with Flag-tagged CD134, ACE2, KREMEN1, ASGR1, CXCR2, or empty. After 24 h, the cells were infected with the GFP-labeled and luciferase SARS-CoV-2 virus pseudotype, and visualized by microscopy (**e**), expression levels of GFP by cell flow cytometer (**f**), and detection of luciferase by microplate (**g**) at 72 h post-infection. Scale bars, 1000 μm. Each data represented the mean ± SD of three independent experiments (*n* = 3) and were analyzed with T-test compared with mock cells. ***p < 0.001

To identify a non-ACE2 SARS-CoV-2 receptor, we established Synergistic Activation Mediator (SAM) library screening^35,36^ in HeLa cells previously shown to be tolerant to SARS-CoV-2 infection due to the inherent low level of ACE2.^15^ This SAM library contains over 70920 gRNAs for 23430 human genes.^35,36^ We prepared MPH lentivirus expressing MS2-p65-HSF1 to infect HeLa cells at a multiplicity of infection (MOI) of 10, after which cells were subjected to hygromycin (200 µg/ml) selection for 14 days (Fig. 1a). We extracted the genome of the selected cell lines and identified it by PCR using primers specifically targeting the MPH plasmid region. We found that infected and drug-screened cell lines had integrated the p65 gene sequence in MPH management (Fig. 1b). We also prepared a SAM library lentivirus to infect HeLa-MPH cells at an MOI of 0.2, aiming to infect single cells with only one virus particle. Then, the cells were selected with blasticidin, and Cas9 expression was analyzed by Western blotting with anti-Cas9 antibody (Fig. 1a, c).

After preparing the HeLa-MPH-SAM cell library, we prepared a pseudovirus containing a Green fluorescent protein (GFP)-luciferase-puromycin construct packaged with wild-type Spike protein to infect the cells from our library at an MOI of 10. Next, puromycin was added to select cells that had been successfully infected (Fig. 1a). After the screening process, we found that most cells had died, whereas the surviving cells grew into single clones. Therefore, we expanded these surviving monoclonal cell populations, extracted the cellular genome, and sequenced the sgRNA sequences present in these monoclonal cells by Sanger sequencing (Fig. 1d). Through statistical analysis, we obtained a total of 13 sgRNAs targeting different genes, including *ACE2*. In addition to *ACE2*, we found candidate genes encoding CD134, KREMEN1, ASGR1, and CXCR2 membrane proteins (Fig. 1d). Considering that SARS-CoV-2 enters cells mainly through the interaction of the wild-type Spike protein and membrane proteins, we focused on the above-mentioned 4 candidate genes for subsequent experiments.

### Validation of candidate genes encoding factors for SARS-CoV-2 pseudovirus entry into cell

To test whether these candidate genes were relevant to SARS-CoV-2 pseudovirus entry into cells,^37,38^ we infected 293 T cells with lentiviruses carrying different candidate genes, followed by clonal selection with puromycin (2 µg/ml) for 14 days. Next, we used wild-type Spike protein-packaged pseudovirus containing GFP and luciferase to infect the cell lines. Using fluorescence microscopy, we found that both positive control 293T-ACE2 cells and 293T-ASGR1 cells were susceptible to infection with SARS-CoV-2 pseudovirus (Fig. 1e). We further confirmed these results through flow cytometry and luciferase reporter gene analysis (Fig. 1f, g). Considering that in the real world, SARS-CoV-2 has undergone multiple evolutions, the current main epidemic strain is XBB.1.16, and in previous epidemics, the delta variant was more pathogenic. Therefore, we also used the Spike protein of XBB.1.16 and B.1.617.2 to package pseudoviruses and infect the above cells, and we found that XBB.1.16 and B.1.617.2 obtained similar results to wild type (Supplementary Fig. 1). Taken together, our data indicates that ASGR1 is a new potential receptor for multiple different SARS-CoV-2 strains.

### SARS-CoV-2 pseudovirus can infect liver cell lines through ASGR1

**Fig. 2:**
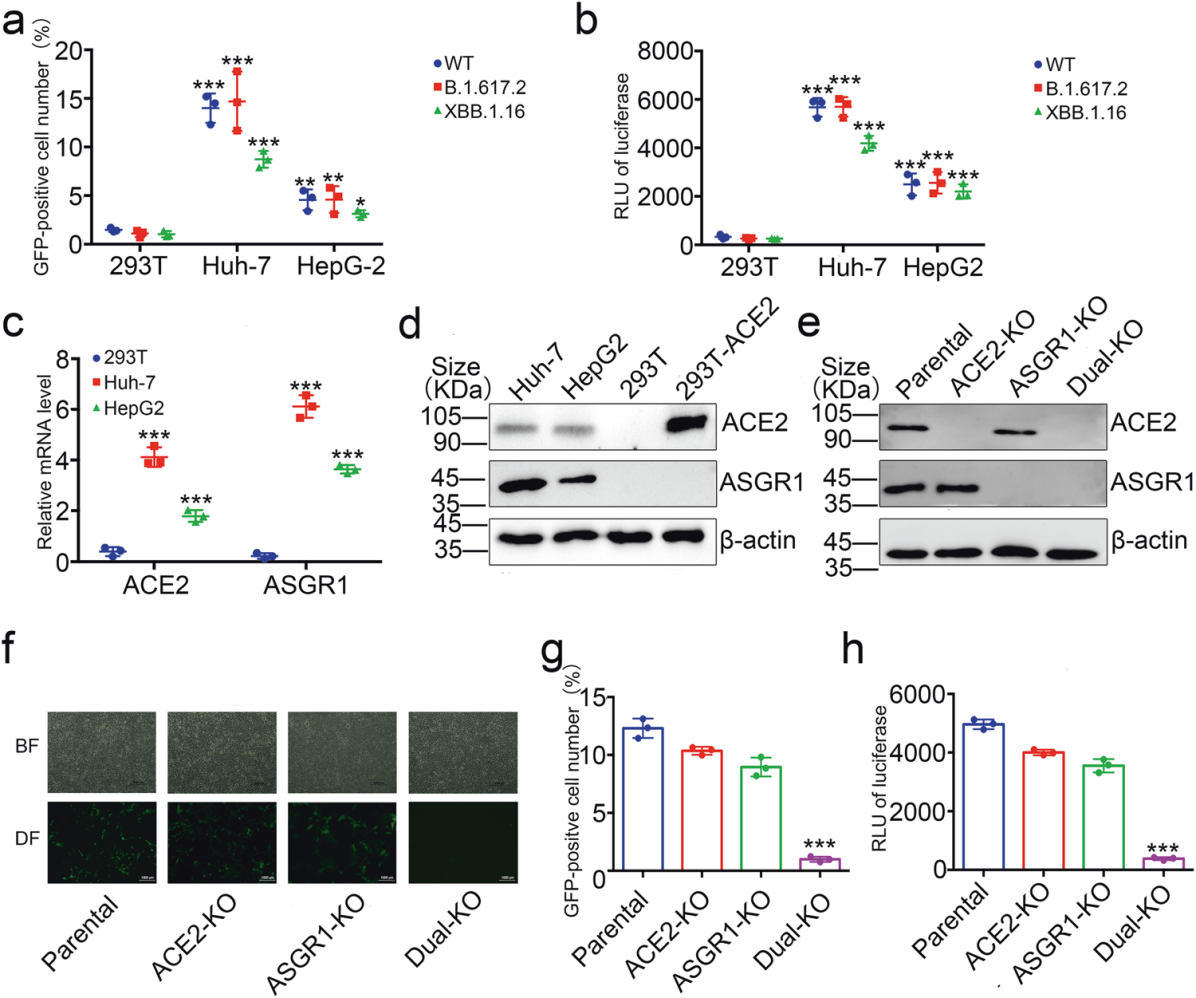
SARS-CoV-2 pseudovirus can infect liver cell lines through ASGR1 in vitro. HEK293T, Huh-7, and HepG2 cells were infected with the GFP-labeled and luciferase SARS-CoV-2 pseudotype virus with the Spike protein of wild type, XBB.1.16 or B.1.617.2, detect the expression levels of GFP by cell flow cytometer (**a**), and luciferase by microplate (**b**) at 72 h post-infection. **c** ACE2 and ASGR1 expressions were measured by qPCR. The intracellular RNA of 293 T, Huh-7, and HepG2 cells was extracted, and after reverse transcription, the corresponding primers were used to amplify ACE2, ASGR1, and GAPDH respectively. The expression levels of ACE2 or ASGR1 were finally normalized by GAPDH. **d** ACE2 and ASSGR1 expressions were measured by Western blot. Western blot of cell lysate of 293T, Huh-7, and HepG2 cells with anti-ACE2 or anti-ASGR1 was performed to identify expression of ACE2 or ASGR1 protein. **e** ACE2 and ASSGR1 expressions were measured by Western blot in Huh-7 cells with knockout of *ASGR1*, *ACE2*, or both with anti-ACE2 or anti-ASGR1. **f**–**h** SARS-CoV-2 pseudovirus with wild-type Spike can infect Huh-7 cells through ACE2 or ASGR1. Huh-7 cells with knockout of *ASGR1*, *ACE2*, or both, were infected with the GFP-labeled and luciferase SARS-CoV-2 pseudotype virus with wild type Spike and visualized by microscopy (**f**), expression levels of GFP by cell flow cytometer (**g**), and detection of luciferase by microplate (**h**) at 72 h post-infection. Scale bars, 1000 μm. Each data represented the mean ± SD of three independent experiments (*n* = 3) and were analyzed with T-test compared with 293 T or parental cells. *p < 0.05; **p < 0.01; ***p < 0.001

Previous studies have shown that ASGR1 is mainly expressed in liver and peripheral blood mononuclear cells.^3,39^ Using the previously constructed SARS-CoV-2 pseudovirus system with reporter gene GFP and luciferase, we found that the pseudovirus could infect Huh-7 and HepG2 cells.^40^ Therefore, we repeated the verification in Huh-7 and HepG2 cells. Through cell flow cytometry and luciferase detection, we found that the wild-type Spike-packaged pseudovirus and the XBB.1.61 or B.1.617.2 Spike-packaged pseudovirus can significantly infect Huh-7 and HepG2 cells. In addition, we found that compared with the wild-type and B.1.617.2 strains, XBB.1.61 infected liver cells decreased to a certain extent (Fig. 2a, b). Results obtained by single-cell sequencing showed that ACE2 expression in human hepatic parenchymal cells was low. Nonetheless, reports still show that ACE2 can be stably expressed in commonly used in vitro liver cell lines.^41^ Therefore, we analyzed the expression of *ACE2* and *ASGR1* mRNAs and the corresponding proteins in Huh-7 cells by qPCR and Western blotting, respectively (Fig. 2c, d). Results showed that both Huh-7 and HepG2 cells expressed ACE2 and ASGR1. However, the expression level of ASGR1 was significantly higher than that of ACE2 (Fig. 2c, d). To further test whether the SARS-CoV-2 pseudovirus entered Huh-7 cells through ASGR1 or ACE2, we knocked out *ASGR1*, *ACE2*, or both by CRISPR/Cas9 (Fig. 2e). Next, we infected those cell lines with SARS-CoV-2 pseudovirus with wild-type Spike and found that knocking out either *ACE2* or *ASGR1* alone did not prevent SARS-CoV-2 pseudovirus from infecting the Huh-7 cells. However, when *ASGR1* and *ACE2* were knocked out simultaneously, the ability of SARS-CoV-2 pseudovirus with wild-type Spike to infect the cells was destroyed, as confirmed by fluorescence microscopy, flow cytometry, and luciferase assays (Fig. 2f–h). These results suggest that two independent receptors may be mediating SARS-CoV-2 infection in Huh-7 cells.

### SARS-CoV-2 pseudovirus infects immortalized hepatocyte lines and primary hepatocytes through ASGR1

**Fig. 3:**
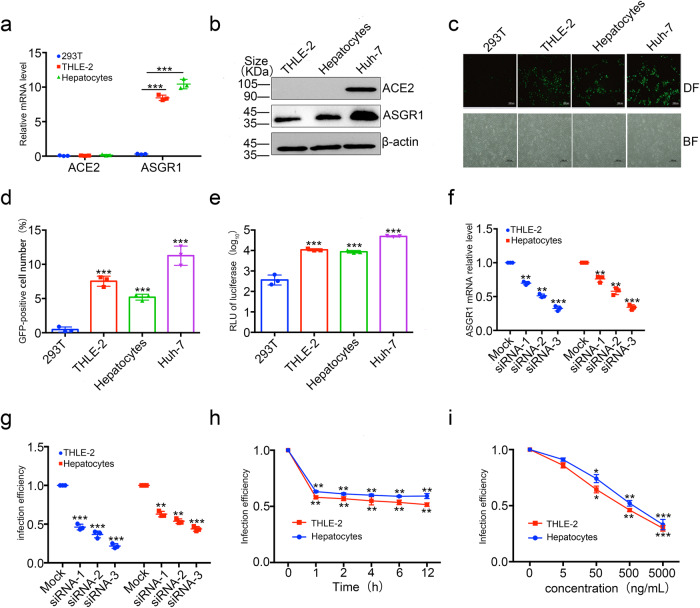
SARS-CoV-2 pseudovirus infects immortalized liver cell lines and primary hepatocytes through ASGR1. **a** ACE2 and ASGR1 expressions were measured by qPCR. The intracellular RNA of 293T, THLE-2, or primary hepatocytes was extracted, and after reverse transcription, the corresponding primers were used to amplify ACE2, ASGR1, and GAPDH respectively. The expression levels of ACE2 or ASGR1 were finally normalized by GAPDH. **b** ACE2 and ASGR1 expressions were measured by Western blot. Western blot of cell lysate of THLE-2, primary hepatocytes, and Huh-7 cells with anti-ACE2 or anti-ASGR1 was performed to identify expression of ACE2 or ASGR1 protein. Each data represented the mean ± SD of three independent experiments (*n* = 3) and were analyzed with T-test compared with 293T cells. **c**–**e** SARS-CoV-2 pseudovirus with wild-type Spike can infect THLE-2 cells and primary hepatocytes. THLE-2 cells and primary hepatocytes were infected with GFP-labeled and luciferase SARS-CoV-2 pseudotype virus with wild type Spike and visualized by microscopy (**c**), the expression levels of GFP by cell flow cytometer (**d**), and detection of luciferase by microplate (**e**) at 72 h post-infection. Scale bars, 1000 μm. Each data represented the mean ± SD of three independent experiments (*n* = 3) and were analyzed with T-test compared with 293 T cells. **f**, **g** siRNA targeting ASGR1 prevents SARS-CoV-2 pseudovirus from infecting liver cells. THLE-2 and primary hepatocytes were treated with 100 nM control-siRNA, siRNA-1, siNRA-2, and siRNA-3 for 24 h, respectively. The intracellular RNA of THLE-2 or primary hepatocytes was extracted, and after reverse transcription, the corresponding primers were used to amplify ASGR1 and GAPDH respectively. The expression levels of ASGR1 were finally normalized by GAPDH. **f**, followed by infection with SARS-CoV-2 pseudotype virus, and infection efficiency was detected by luciferase (**g**). Each data represented the mean ± SD of three independent experiments (*n* = 3) and were analyzed with T-test compared with Mock cells. **h**, **i** ASGR1 monoclonal antibody can prevent SARS-CoV-2 pseudovirus from infecting liver cells. THLE-2 cells and primary hepatocytes were treated with 500 μg/mL anti-ASGR1 antibody for 0 h, 1 h, 2 h, 4 h, 8 h, and 12 h (h) or with 0 μg/mL, 5 μg/mL, 50 μg/mL, 500 μg/mL, and 5000 μg/mL for 6 h (**i**) and then infected with SARS-CoV-2 pseudotype virus, and the infection efficiency was detected by luciferase. Each data represented the mean ± SD of three independent experiments (*n* = 3) and were analyzed with T-test compared with 0 h or 0 μg/mL treated with cells. *p < 0.05; **p < 0.01; ***p < 0.001

Given that the expression level of ACE2 in normal liver tissue is much lower than 0.1%,^16–18^ while the expression level of ACE2 in liver tumor cell lines, such as HepG2 and Huh-7, is relatively high, the latter cannot fully reflect the condition of hepatic parenchymal cells in the human body. Therefore, we further explored the effect of SARS-CoV-2 pseudovirus infection on the immortalized hepatocyte cell line THLE-2 and primary hepatocytes. We first determined the expression levels of ACE2 and ASGR1 proteins in THLE-2 cells and primary hepatocytes. Unlike Huh-7 and HepG-2 liver tumor cell lines, THLE-2 cells and primary hepatocytes expressed nearly no ACE2, whereas ASGR1 was highly expressed (Fig. 3a, b), suggesting, in turn, that these two cell types may be a suitable cell system for the study of SARS-CoV-2 receptors. Accordingly, we first infected THLE-2 cells and primary hepatocytes with SARS-CoV-2 pseudovirus with wild-type Spike and found that SARS-CoV-2 pseudovirus could infect both cell types (Fig. 3c–e).

Given the short culture cycle of primary cells, it is not suitable to screen cells through CRISPR-Cas9-dependent knockout. Therefore, in THLE-2 cells and primary hepatocytes, we knocked down ASGR1 receptors with siRNA and blocked ASGR1 binding using ASGR1 monoclonal antibody to determine whether SARS-CoV-2 pseudovirus infection could be prevented. In siRNA-treated cells, the expression of ASGR1 significantly decreased (Fig. 3f). Correspondingly, the proportion of cells infected with SARS-CoV-2 pseudovirus also decreased significantly (Fig. 3g). For comparison, we treated THLE-2 cells with siRNA targeting ACE2, and detected ACE2 mRNA levels by qPCR. It was found that no matter which siRNA was used, the ACE2 level of THLE-2 could not be significantly reduced (Supplementary Fig. 2a). Considering that its CT value was close to 35, we believed that the siRNA knockdown effect was not obvious because the background ACE2 level of THLE-2 was too low. Therefore, we further repeated the knockdown experiment of siRNA targeting ACE2 in Huh-7 cells and found that all the designed siRNA could significantly knock down the mRNA level of ACE2 (Supplementary Fig. 2b). After that, we used pseudoviruses with wild-type Spike protein to infect THLE-2 cells with ACE2 knockdown via siRNA targeting, and we found that the level of pseudovirus infection was not significantly reduced (Supplementary Fig. 2c). Then, we observed that similar to cells treated with siRNA targeting ASGR1, cells treated with monoclonal anti-ASGR1 also showed a decrease in the level of SARS-CoV-2 infection (Fig. 3h, i), noting that monoclonal antibodies are not as effective in inhibiting viral infection as siRNA.

Considering that under physiological conditions, ASGR1 can promote the endocytosis of glycoproteins containing N-acetyl galactosamine residues by binding to N-acetyl galactosamine,^42^ we tried to further explore whether N-acetyl galactosamine blocks the infection of hepatocytes by SARS-CoV-2. We treated THLE-2 cells and hepatocytes with different concentrations of N-acetyl galactosamine and simultaneously infected cells with pseudoviruses containing wild-type Spike protein. It was found that at high concentrations (10–100 mM), N-acetyl galactosamine could inhibit SARS-CoV-2 pseudovirus infection to a certain extent (Supplementary Fig. 3), but its effect was not as good as that of siRNA or ASGR1 monoclonal antibody. These results indicate that ASGR1 is the main receptor for SARS-CoV-2 pseudovirus infection in immortalized and primary hepatocytes.

### SARS-CoV-2 infects primary hepatocytes through ASGR1 and ACE2 in non-alcoholic fatty liver disease

Considering that some of the patients with SARS-CoV-2 infection detected in the liver had non-alcoholic fatty liver disease,^7,10^ we are also very curious about the infection of hepatocytes by SARS-CoV-2 in the context of non-alcoholic fatty liver disease. First, we constructed a non-alcoholic fatty liver cell model using THLE-2 cells according to the literature.^43^ The hepatocytes we detected earlier do not express ACE2; however, studies have shown that the expression level of ACE2 is increased in hepatocytes of patients with liver diseases. Therefore, we first detected the expression of ASGR1 and ACE2 on the constructed non-alcoholic fatty liver disease cell model. It was found that compared with normal THLE-2 cells, the mRNA level of ACE2 was significantly increased in THLE-2 cells of the non-alcoholic fatty liver disease cell model, but ASGR1 was not significantly changed (Supplementary Fig. 4a). After that, we used pseudoviruses containing Spike proteins of different strains to infect normal THLE-2 and the non-alcoholic fatty liver disease cell model. We found that in the non-alcoholic fatty liver disease cell model, whether it was the wild-type Spike protein pseudovirus or the B.1.617.2 or XBB.1.16 strain, the infection level was significantly increased, consistent with the increase in the expression level of ACE2 (Supplementary Fig. 4a–c). The result suggests that in non-alcoholic fatty liver disease, SARS-CoV-2 can infect hepatocytes through ASGR1 and ACE2.

### SARS-CoV-2 infects primary hepatocytes through ASGR1

**Fig. 4:**
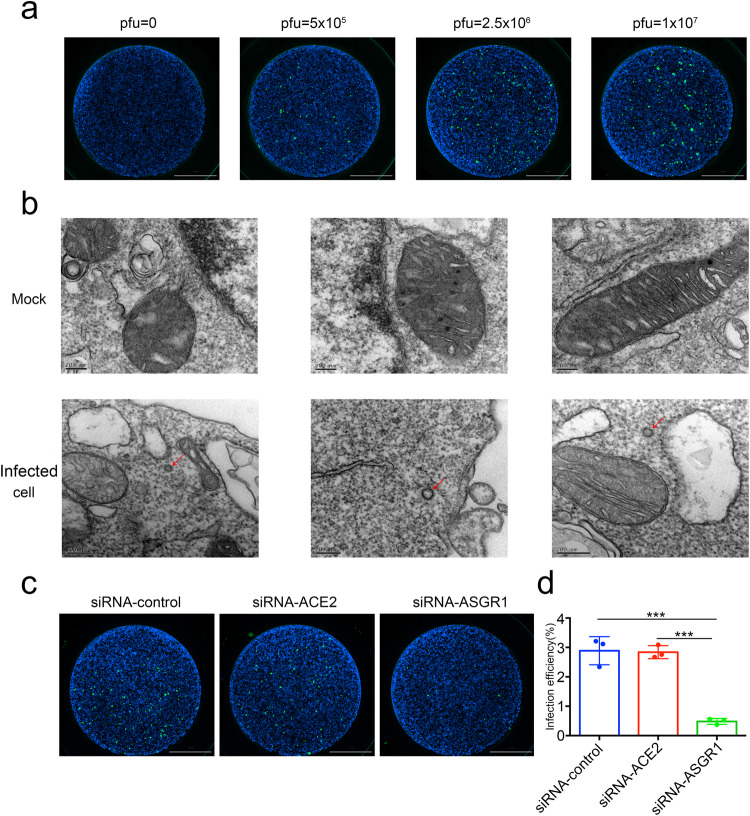
SARS-CoV-2 with wild-type Spike infects primary hepatocytes through ASGR1. **a** Primary hepatocytes were infected with SARS-CoV-2 with wild-type Spike at different pfu (pfu = 0, 5.0 ×105, 2.5 ×106, or 1 ×107) for 6 h and replaced the virus-free complete medium to continue culturing for 24 h. Then cells were immunostained with rabbit-anti-SARS-CoV-2 NP and DAPI. **b** Morphological detection of SARS-CoV-2 in primary hepatocytes. After cells were infected with SARS-Cov-2 with wild-type Spike and fixed with methanol overnight, they were negatively stained and observed using a projection electron microscope. **c**, **d** siRNA targeting ASGR1 prevented SARS-CoV-2 with wild-type Spike from infecting liver cells. Primary hepatocytes were treated with control-siRNA, siRNA-ACE2, or siRNA-ASGR1 for 24 h, respectively. Then primary hepatocytes were infected with SARS-CoV-2 with wild-type Spike (pfu = 1 ×107) for 6 h and replaced the virus-free complete medium to continue culturing for 24 h. After that, the primary hepatocytes were immunostained with rabbit-anti-SARS-CoV-2 NP and DAPI (**c**). The intensity of NP were quantified using Image J and shown in **d**, which represents the infection efficiency of the SARS-CoV-2. Each data represented the mean ± SD of three independent experiments (*n* = 3) and were analyzed with T-test compared with cells treated with siRNA. ***p < 0.001

To account for differences between pseudovirus and live SARS-CoV-2, we further confirmed whether SARS-CoV-2 could infect primary hepatocytes through ASGR1. We first infected primary hepatocytes with wild-type, XBB.1.16 or EG5 SARS-CoV-2^44^ at different pfu for 6 h and detected SARS-CoV-2 in the cells by immunofluorescence. Results confirmed the presence of N protein in some primary hepatocytes (Fig. 4a and Supplementary Fig. 5a), suggesting that some primary hepatocytes had been infected by SARS-CoV-2. After that, transmission electron microscopy confirmed that SARS-CoV-2 virus does exist in hepatocytes (Fig. 4b). To further confirm that SARS-CoV-2 infects primary hepatocytes through ASGR1, we first knocked down ASGR1 with siRNA. After 24 h, wild-type, XBB.1.16 or EG5 SARS-CoV-2 was added and incubated for 6 h, followed by immunofluorescence detection. Compared with the control-siRNA and ACE2-knockdown group, ASGR1-knockdown could significantly reduce the infection of hepatocytes by SARS-CoV-2 (Fig. 4c, d and Supplementary Fig. 5b, c). Thus, in primary hepatocytes, ASGR1 is the main infection receptor of SARS-CoV-2.

### ASGR1 could bind to the RBD and NTD regions of Spike protein and infection dependent on RBD

In order to further confirm that ASGR1 is a potential receptor of SARS-CoV-2, we transiently transfected ASGR1-FLAG or ACE2-FLAG and Spike-HA plasmids into 293 T cells and found that there was a significant interaction between ASGR1 and Spike proteins (Supplementary Fig. 6a). In addition, we also used His-tagged Spike protein as the primary antibody, and detected it by flow cytometry in 293 T cells transfected with empty plasmid, FLAG-ASGR1 or FLAG-ACE2. It was found that in 293 T cells transfected with empty plasmid, His-tagged Spike could not bind to the cells; however, no matter whether the cells transfected with FLAG-ASGR1 or FLAG-ACE2, His-tagged Spike could bind to cells, and the binding efficiency of His-tagged Spike with 293 T cells transfected with FLAG-ACE2 is higher than that of 293 T cells transfected with FLAG-ASGR1 (Supplementary Fig. 6b). Then, in order to explore the region where ASGR1 binds to the Spike protein, we used ELISA experiments to co-incubate with different concentrations of ASGR1 and different Spike regions, and found that ASGR1 mainly interacts with the RBD and NTD and the binding affinity of ASGR1 and NTD is higher than that of RBD (Kd_(NTD)_ = 178.9 nM; Kd_(RBD)_ = 269.4 nM) (Supplementary Fig. 6c). Given that ACE2 mainly binds to the RBD region, we infer that ASGR1 may compete with ACE2 for binding to RBD. Therefore, we conducted competitive binding experiments by ELISA and found that ASGR1 could indeed destroy the binding of ACE2 and RBD (Supplementary Fig. 6d). Later, in order to further confirm whether SARS-CoV-2 infects cells through ASGR1 mainly through the NTD region or the RBD region, we used excess RBD or NTD protein to treat 293T-ASGR1 in advance, and then infected it with SARS-CoV-2 pseudovirus. The results showed that compared with NTD protein, when cells were pretreated with RBD protein, the infection efficiency of SARS-CoV-2 pseudovirus was significantly reduced (Supplementary Fig. 6e, f). This result prompted us that although the binding affinity of ASGR1 and NTD regions is higher, SARS-CoV- 2 still mainly relies on the RBD region to infect cells through ASGR1. Taken together, we confirm that ASGR1 could bind to the RBD and NTD regions of Spike protein and the infection dependent on RBD.

### Soluble ASGR1 protein can prevent SARS-CoV-2 pseudovirus from infecting various cells

**Fig. 5:**
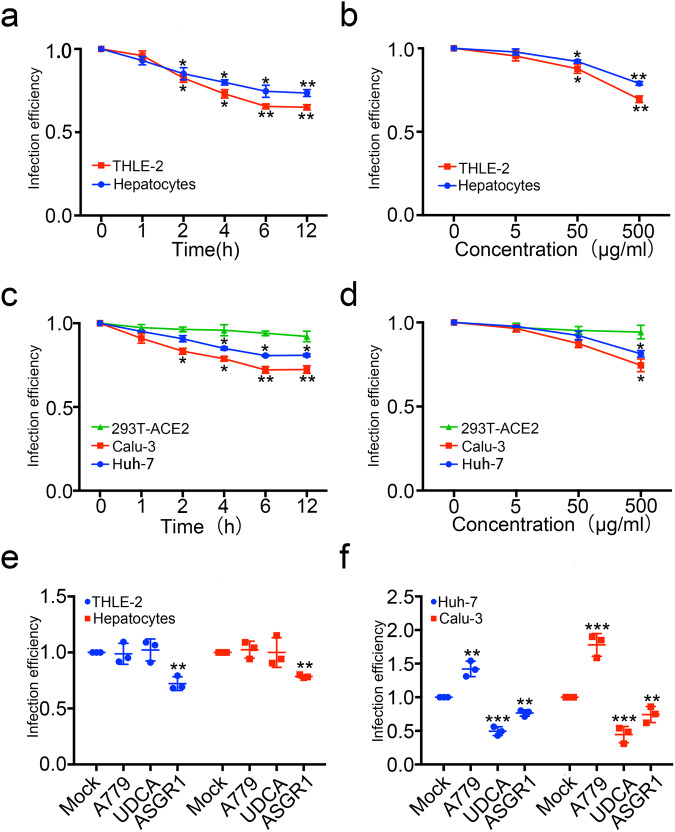
Soluble ASGR1 protein can prevent SARS-CoV-2 pseudovirus from infecting a variety of cells. **a**, **b** Soluble ASGR1 protein could prevent SARS-CoV-2 pseudovirus from infecting liver cells. The SARS-CoV-2 pseudotype virus with wild type-Spike was incubated with 500 μg/mL soluble ASGR1 for 0 h, 1 h, 2 h, 4 h, 8 h, and 12 h (**a**) or with 0 μg/mL 5 μg/mL, 50 μg/mL and 500 μg/mL for 6 h (**b**), then infected THLE-2 cells and primary hepatocytes. The infection efficiency was detected by luciferase. **c**, **d** Soluble ASGR1 protein could prevent SARS-CoV-2 pseudovirus from infecting cells expressing ACE2. The SARS-CoV-2 pseudotype virus with wild-type Spike was incubated with 500 μg/mL soluble ASGR1 for 0 h, 1 h, 2 h, 4 h, 8 h, and 12 h (**c**) or with 0 μg/mL 5 μg/mL, 50 μg/mL and 500 μg/mL for 6 h (**d**), then infected Huh-7, Calu-3 and 293T-ACE2 cells. The infection efficiency was detected by luciferase. **e** Comparison of ACE2 inhibitors and ASGR1 on SARS-CoV-2 pseudovirus-infected cells. Before infection of THLE-2 cells and primary hepatocytes (**e**) or Huh-7 and Calu-3 (**f**), the SARS-CoV-2 pseudotype virus with wild type-Spike was incubated with 500 μg/mL soluble ASGR1 for 12 h or THLE-2 cells and primary hepatocytes (**e**) or Huh-7 and Calu-3 (**f**) treated with A779 (10 μM) or UDCA (10 μM) for 12 h. The infection efficiency was detected by luciferase. Each data represented the mean ± SD of three independent experiments (*n* = 3) and were analyzed with T-test compared with 0 h or 0 μg/mL treated with cells. *p < 0.05; **p < 0.01; ***p < 0.001

After confirming that ASGR1 is a potential new receptor for SARS-CoV-2 and considering that there is a competitive binding relationship between ASGR1 and ACE2, we tested whether ASGR1 protein could be used for the prevention of SARS-CoV-2 pseudovirus infections. We first obtained soluble ASGR1 protein using 293 T cells by transfection with an expression plasmid, followed by immunoprecipitation-based purification (Supplementary Fig. 7). After that, we co-incubated soluble ASGR1 protein and SARS-CoV-2 pseudovirus and then infected THLE-2 and primary hepatocytes. As the concentration of soluble ASGR1 protein and time of co-incubation were increased, the efficiency of wild-type and B.1.617.2, XBB.1.16 SARS-CoV-2 pseudovirus infection gradually decreased (Fig. 5a, b and Supplementary Fig. 8a, b). In addition to hepatocytes, we asked if soluble ASGR1 could prevent susceptible cells featuring ACE2-mediated entry from being infected by SARS-CoV-2 pseudovirus. To address this question, we repeated the experiment with Calu-3, 293T-ACE2, and Huh-7 cells and found that infection efficiencies in Calu-3 and Huh-7 cells were significantly decreased, while the decrease was not as significant in 293T-ACE2 cells (Fig. 5c, d and Supplementary 8c, d). To account for this, we speculate that the expression level of ACE2 in 293T-ACE2 cells was assumed to be much higher than that under physiological conditions, thus potentially obscuring the treatment effects.

After that, we further compared the ASGR1 soluble protein with two ACE2 inhibitors that have been reported so far—A779^45^ and ursodeoxycholic acid (UDCA).^46^ We selected the best effects of the three for comparison. It was found that neither A779 nor UDCA could inhibit the infection of SARS-CoV-2 in THLE-2 or primary hepatocytes, but soluble ASGR1 protein could (Fig. 5e). In Huh-7 and Calu-3 cells expressing ACE2, both soluble ASGR1 protein and UDCA could significantly inhibit SARS-CoV-2 infection, but the inhibitory effect of soluble ASGR1 protein was not as good as UDCA. However, A779 could promote infection, which is consistent with previous reports (Fig. 5f). Finally, considering that SARS-CoV-2 mainly infects epithelial cells of the respiratory tract, we decided to explore whether soluble ASGR1 protein would have an effect on the proliferation and apoptosis of respiratory epithelial cells. Irrespective of concentration, results showed that soluble ASGR1 had no significant effect on the proliferation and apoptosis of BEAS-2B lung epithelial cell line (Supplementary Fig. 9).

## Discussion

SARS-CoV-2 mainly infects respiratory system cells, but increasing evidence reveals that it can infect almost all major body organs, including the heart, brain, and liver.^3,7–10^ Initially, researchers detected virus particles in epithelial cells of the respiratory tract, but recently, SARS-CoV-2 virus particles were found in pathology sections of various organs, such as kidney, liver, and duodenum.^3,7–10^ Clinical data show that more than 50% of COVID-19 patients have severe liver damage and inflammation.^11–13^ In addition, SARS-CoV-2 particles were also observed through electron microscopy in hepatic parenchymal cells of COVID-19 patients’ autopsies,^3–9^ which suggests that SARS-CoV-2 infection of hepatocytes may be the cause of severe liver damage in patients. However, the first-discovered SARS-CoV-2 receptor ACE2 is highly expressed in epithelial cells of the respiratory and digestive tracts, whereas its expression in hepatic parenchymal cells is 0.1%.^16–18^ Current reports show that ACE2 expression levels are high in intrahepatic cholangiocytes. Given that the main feature of the patients with liver injury clinically is elevated transaminase activity rather than cholestasis, functionally impaired cells should theoretically be hepatocytes. Therefore, the existence of non-ACE2 SARS-CoV-2 receptors in hepatocytes cannot be ruled out.

Many studies have reported on new receptors for SARS-CoV-2, but most receptors are co-receptors for ACE2, such as host proteases transmembrane proteases serine 2 (TMPRSS2)^14^ and Neuropilin-1 (NRP-1).^8,22^ The only known non-ACE2 receptors are AXL^21^ and CD147.^24^ However, AXL and CD147 are mainly expressed in lung cells and monocytes, respectively, which cannot explain the infection of other tissues by SARS-CoV-2. On the other hand, more information about the SARS-CoV-2 receptor can be found in some preprinted studies that have not been peer-reviewed.^19,20,25,26^ However, these receptor screenings are mainly concentrated in a few tool cells, such as A549 and Calu-3, while ignoring the physiological significance of the existence of non-ACE2 receptors of SARS-CoV-2.^23,25,26^

In this study, we conducted a gain-of-function screening experiment for the entry of SARS-CoV-2 into host cells and identified ASGR1 as the main receptor for SARS-CoV-2 entry into hepatocytes. Previous studies confirmed that ASGR1 could promote HBV to infect human hepatocytes.^47^ Moreover, owing to its high expression in hepatic parenchymal cells, ASGR1 is one of the current candidate targets for the treatment of liver cancer.^38,48^ Previously, Gu et al. reported that ASGR1 is a potential receptor of SARS-CoV-2, but they only proved that the overexpression of ASGR1 in non-hepatocytes promoted SARS-CoV-2 infection, and did not explain the physiological role of ASGR1 during SARS-CoV-2 infection.^23^ Besides, Collins et al. Also found that there is an interaction between ASGR1 and Spike protein, but they also did not explain the significance of the interaction between ASGR1 and Spike.^49^ Therefore, before the discovery of our study, it was unclear exactly whether and how SARS-CoV-2 infected hepatocytes.

Compared with ACE2, conclusive evidence from the analysis of patient-derived liver samples from COVID-19 autopsies^7,9^ shows that ASGR1, highly expressed in hepatocytes, is more likely to be the main receptor for SARS-CoV-2 entry. In addition, we found that the knockdown of ASGR1 with siRNA could significantly reduce SARS-CoV-2 infection in primary hepatocytes. In addition, soluble ASGR1 protein not only inhibited SARS-CoV-2 from infecting a liver cell line, but also prevented SARS-CoV-2 from infecting cells expressing ACE2 receptors, such as Calu-3 and Huh-7 cells.

Taken together, our findings demonstrate that ASGR1 plays an indispensable role in facilitating SARS-CoV-2 entry into hepatocytes. Therefore, upon further validation and mechanistic elucidation, we anticipate that our findings will contribute to the development of therapeutic solutions for SARS-CoV-2 infection.

## Materials and methods

### Ethics statement

This study was approved by the Ethics Committee of School of Life Sciences, Fudan University and the methods were consistent with the relevant guidelines and regulations of that committee (BE2001). Primary hepatocytes were donated from healthy individuals, and the donors were informed and consented to the contents of this study.

### Antibody and reagents

The following antibodies were used throughout this study: from Abclonal (Wuhan, China), anti-FLAG (AE063), anti-HA (AE036), anti-ASGR1 (A13279), anti-β-Actin (AC026). From Abcam (Cambridge, UK), anti-ASGR1 (ab254262). From proteintech (Wuhan, China), anti-ACE2 (21115-1-AP). From Sino Biological (Beijing, China), rabbit-anti-SARS-CoV-2 NP antibody. From R&D systems (UMN, USA), anti-His-Alexa Fluor488-conjugated Antibody (IC050G). Anti-Flag Magnetic Beads (HY-K0207) and ACE2/ANG (1-7) inhibitor-A779 (HY-P0216) were purchased from MCE. The Sipke of SARS-CoV-2 (DRA59) was purchased from novoprotein (Shanghai, China). 2× Taq Master Mix (P112) and High-fidelity PCR enzyme-2× Phanta Max Master Mix (P515) were purchased from Vazyme (Nanjing, China). PMD18-T (6011) was purchased from Takara (Beijing, China). Cell Genome Extraction Kit (DP304) and Plasmid Extraction Kit (DP103, DP108, DP117) were purchased from Tiangen (Beijing, China). Gel Extraction Kit (CW2302) was purchased from CWBIO (Nanjing, China). Luciferase detection kit (E6110) was purchased from Promega (Madison, USA). Cell Counting Kit (CCK-8) and TUNEL Apoptosis Detection Kit (FITC) were purchased from Yeasen (Shanghai, China).

### Cell culture and virus

293T, HeLa, HepG-2, Huh-7, Calu-3, and BEAS-2B cells were cultured in DMEM (Gibco, C11995500BT) with 10% fetal bovine serum (FBS) (ExCell Bio, FSP500) and 1% penicillin/streptomycin (P/S) (Gibco, 15140-122) in a 37 °C incubator containing 5% CO2. THLE-2 cells were exposed to odium oleate (OA) dissolved in 0.5% fatty acid-free bovine serum albumin (BSA) at a final concentration of 0.3 mmol/L for 24 h to establish an in vitro non-alcoholic fatty liver disease model. THLE-2 and primary hepatocytes were cultured in BEGM (Lonza, CC-3170) in 5 ng/mL EGF, 70 ng/mL Phosphoethanolamine, and 10% FBS in a 37 °C incubator containing 5% CO2. The SARS-CoV-2 strain (GenBank accession number: 622319) was isolated from a laboratory-confirmed COVID-19 patient. All experiments involving live SARS-CoV-2 were performed in a Biosafety Level 3 (BLS-3) laboratory of the Navy Medical University.

### Pooled genome-wide CRISPR screen

A total of 1 × 107 HeLa cells were infected with MPH lentivirus (MOI = 10). After 72 h, HeLa cells were selected with 100 μg/ml Hygromycin B for 14 days. Then, a total of 1 × 109 HeLa cells expressing MPH were infected at a low multiplicity of infection (MOI = 0.2) to ensure that most cells received only one viral construct. After 72 h, the cells were selected with 2 μg/ml Blasticidin for 14 days.

### Vector construction, Cas9-mediated gene knockout, and cDNA overexpression

Individual sgRNA constructs targeting ASGR1 or ACE2 were cloned into lentiCRISPR v2.0 (addgene 52961). For cDNA expression vectors, a linearized lentiviral backbone was generated from PCDH (Youbio, Hunan, China). Protein-coding sequences were a gift from MiaoLing Plasmid Sharing Platform. All the constructed plasmids were confirmed by restriction enzyme digestion and DNA sequencing. Huh-7 or 293T cells were infected with lentivirus at an MOI of 1 and then selected using 2 μg/ml puromycin for 14 days for knockout or overexpression. Knockout efficiency was analyzed using Sanger DNA sequencing. Knockout efficiency was detected by Western blot (WB) analysis.

### Construction and production of pseudoviruses

The spike of SARS-CoV-2 and that of all variant plasmids were synthesized by GeneScript. All S proteins were optimized with 18 amino acids removed and an HA tag attached. The core plasmid, pLenti.GFP.NLuc-Puromycin, which expresses GFP, luciferase, and puromycin at the same time was maintained in our laboratory.

For the production of pseudoviruses, HEK293T/17 cells were seeded into 10 cm dishes one day before transfection. When the cells reached 80% confluence, plasmid and PEI were added into Opti-MDM (Gibco), mixed evenly, and left standing for 20 min. pLenti.GFP.NLuc, psPAX2, and the S protein vector were co-transfected into HEK293T/17 cells to produce the pseudoviruses. After incubation at 37 °C and 5% CO2 for 24 h, the culture medium was changed with DMEM with 2% FBS and 1% P/S. The supernatants containing SARS-CoV-2 pseudotyped viruses were harvested at 48 h and 72 h after transfection and filtered by 0.45μm pore size. The filtrates were centrifuged at 25,000 rpm and 4 °C for 2 h. The supernatants were discarded, and the pseudovirus stocks were dissolved in Lentivirus Freezing Solution (AC04L452, Life-iLab, Shanghai) for storage at −80 °C.

### Visualization of GFP and flow cytometry assay

For EGFP expression, Cells were collected and washed with phosphate-buffered saline (PBS). They were kept in PBS before analysis on a BD LSRII flow cytometer. For the binding experiment between Spike protein and 293T cells expressing ASGR1 or ACE2, after collecting the cells, use 10 ug/ ml of Spike of SARS-CoV-2 to incubate with the cells for 30 min, washed with PBS 3 times, and use His-Alexa Fluor488-conjugated Antibody Incubate for 30 min. Then, cells were kept in PBS before analysis on a BD LSRII flow cytometer. FlowJo software (FlowJo LLC, Ashland, OR) was used to perform flow cytometry analysis.

### Immunofluorescence assay

After cells were infected with SARS-CoV-2, they were fixed with methanol. After blocking with 3% bovine serum albumin for 2 h, the cells were incubated overnight at 4 °C with a rabbit-anti-SARS-CoV-2 NP antibody (Sino Biological). Then, the cells were incubated with Alexa Fluor 488-conjugated secondary antibody (Thermo Fisher Scientific) for 2 h at room temperature. Finally, phosphate-buffered saline (PBS) supplemented with 0.1 μg/ml DAPI (Sigma-Aldrich) was added to the cells for 15 min. Images were acquired using Cytation 5 (Biotek). All acquired data were analyzed by Image J.

### Luciferase reporter assay

Cells were collected and washed with phosphate-buffered saline (PBS). Cells were harvested at 72 h post-infection, and the lysate was assayed for luciferase activity. Triplicate cultures were measured for each experiment.

### Western blot

A total of 1 × 10^6^ cells were preseeded in a 10-cm dish and cultured for 24 h. Then cells were harvested, lysed, subjected to SDS PAGE, and then transferred on N.C membrane, followed by incubation with indicated primary antibody. Membranes were visualized using the Immun-Star WesternC Chemiluminescence Kit (Bio-Rad), and images were captured using a ChemiDoc XRS+ System and processed using ImageLab software (Bio-Rad).

### Protein purification

ASGR1 protein was produced in HEK 293T cells. After the plasmid of FLAG-ASGR1 protein was transfected for 48 h, cells were collected and lysed. Anti-Flag protein A/G magnetic beads were then used for immunoprecipitation and quantified by BCA kit.

### Binding of ASGR1 and Spike protein

ELISA was used to detect the binding of ASGR1 and Spike protein. The plate was coated with 100 μl per well of RBD, NTD, or S2 protein (Novoprotein). The plate was sealed and incubated overnight at room temperature. After blocking, the plate was incubated at room temperature for 1 h. The purified ASGR1-HA protein diluted in reagent diluent was added and incubated for 2 h. After washing,100 μl of the working dilution of anti-HA were added to each well. Another incubation was performed for 2 h in which goat anti-mouse IgG HRP was combined with anti-HA to reveal the binding of ASGR1 and Spike protein with the addition of substrate.

### Cell proliferation by CCK-8 assay

Five thousand cells were added to each well of a 96-well plate. To measure the OD value, 10% CCK8 solution was added to fresh culture medium and incubated at 37 °C for 1 h. The OD 450 nanometer value was measured.

### Apoptosis detected by TUNEL staining

A total of 1 × 106 cells were collected in a 1.5 ml tube and centrifuged at 300 g for 5 min. Cells were washed twice with 500 μL PBS. Cells were treated with TUNEL-FITC apoptosis detection kit according to the manufacturer’s instructions. The proportion of FITC-positive cells was analyzed by flow cytometer. FlowJo software (FlowJo LLC, Ashland, OR) was used to perform flow cytometry analysis.

### Statistical analysis

Data are representative of three independent experiments, and error bars represent standard errors (SD). Paired samples t-tests were performed with the use of SPSS version 13.0 (SPSS Inc., Chicago, IL, USA), and statistical significance was indicated at *P < 0.05, **P < 0.01 or ***P < 0.001.

### Supplementary information

Supplementary Materials

## Data Availability

The data associated with this paper are available upon request to the corresponding author.
