# Supplementary material for: Asialoglycoprotein receptor 1 promotes SARS-CoV-2 infection of human normal hepatocytes
Source: Signal Transduct Target Ther. 2024 Feb 14;9:42. doi: 10.1038/s41392-024-01754-y (PMC10866945; doi:10.1038/s41392-024-01754-y)
Supplement: Supplementary file 1 — Supplementary Materials [file 41392_2024_1754_MOESM1_ESM.pdf]

Supplementary Materials for

Asialoglycoprotein receptor 1 promotes SARS-CoV-2 infection of human normal hepatocytes

Xinyi Yang[1,8], Xu Zheng[2,8], Yuqi Zhu[1], Xiaying Zhao[1], Jun Liu[1], Jiangna Xun[1,3], Songhua Yuan[3], Jun Chen[3,4], Hanyu Pan[1], Jinlong Yang[1], Jing Wang[1], Zhimin Liang[1], Xiaoting Shen[1], Yue Liang[1], Qinru Lin[1], Huitong Liang[1], Min Li[1], Fei Peng[7], Daru Lu[1], Jianqing Xu[5], Hongzhou Lu[3,4,6], Shibo Jiang[5] , Ping Zhao[2*], Huanzhang Zhu[1,*]

Correspondence to: Ping Zhao (pnzhao@163.com) and Huanzhang Zhu (hzzhu@fudan.edu.cn)

This PDF file includes:

Figures. S1 to S10

Supplementary Figures and Figure Legends

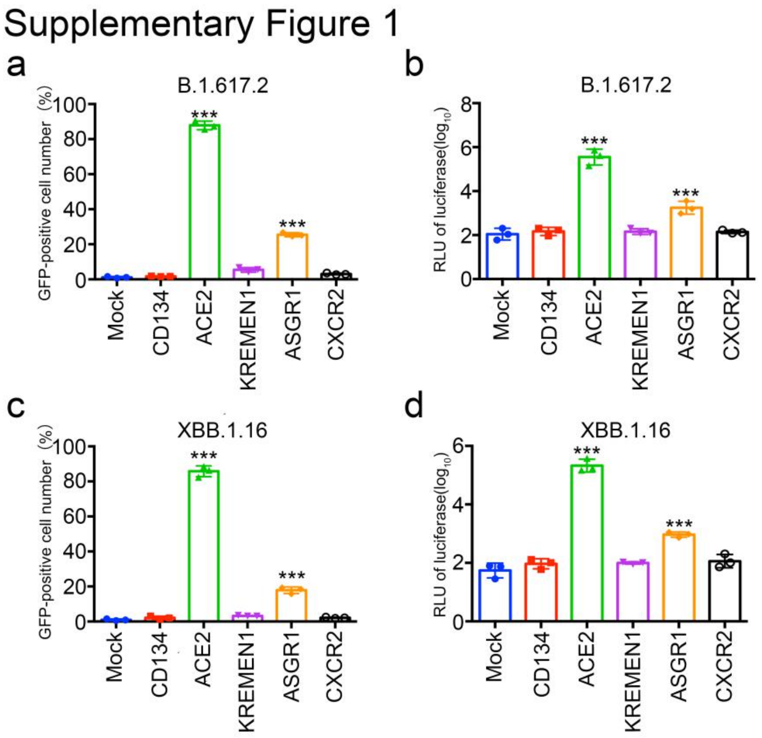

Supplementary Figure S1. ASGR1 facilitates SARS-CoV-2 virus pseudotype with Spike of B.1.617.2 and XBB.1.16 infection as potently as ACE2.

(a-d) HEK293T cells were transfected with Flag-tagged CD134, ACE2, KREMEN1, ASGR1, CXCR2, or empty. After 24 h, the cells were infected with the GFP-labeled and luciferase SARS-CoV-2 virus pseudotype with Spike of B.1.617.2 **(a,b)** or XBB.1.16 **(c,d)**, then detection of the expression levels of GFP by cell flow cytometer **(a,c)** and luciferase by microplate **(b,d)** at 72 h post-infection. Each data represented the mean ± SD of three independent experiments (n=3) and were analyzed with T-test compared with mock cells. ***, $p < 0.001$.

Supplementary Figure 2

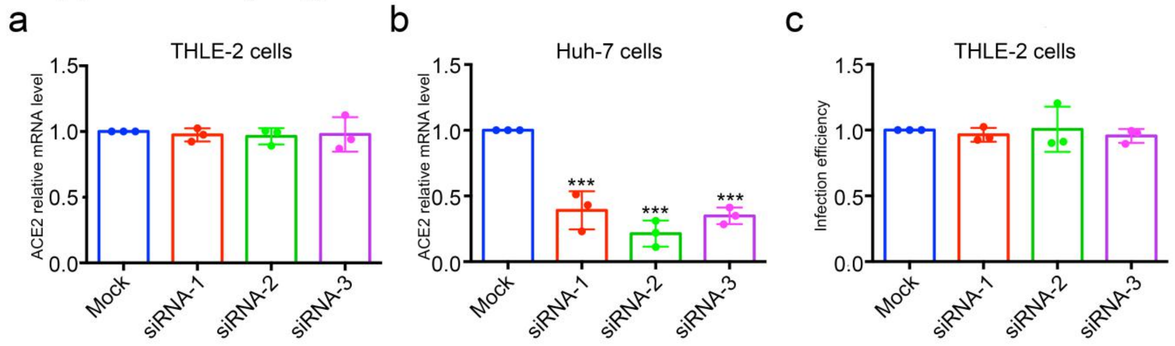

Supplementary Figure S2. siRNA targeting ACE2 does not affect infection of hepatocytes by SARS-CoV-2 pseudovirus.

(a,b) THLE-2 cell or Huh-7 was treated with 100nM control-siRNA, siRNA-1, siNRA-2 and siRNA-3 for 24 h, respectively. The intracellular RNA of THLE-2 or Huh-7 cell was extracted, and after reverse transcription, the corresponding primers were used to amplify ACE2 and GAPDH respectively. The expression levels of ACE2 were finally normalized by GAPDH. **(c)** THLE-2 cells which were treated with 100 nM control-siRNA, siRNA-1, siNRA-2, and siRNA-3 for 24 h, respectively, were infected with SARS-CoV-2 pseudotype virus with wild-type Spike, and infection efficiency was detected by luciferase. Each data represented the mean ± SD of three independent experiments (n=3) and were analyzed with T-test compared with mock cells. ***, $p < 0.001$.

Supplementary Figure 3

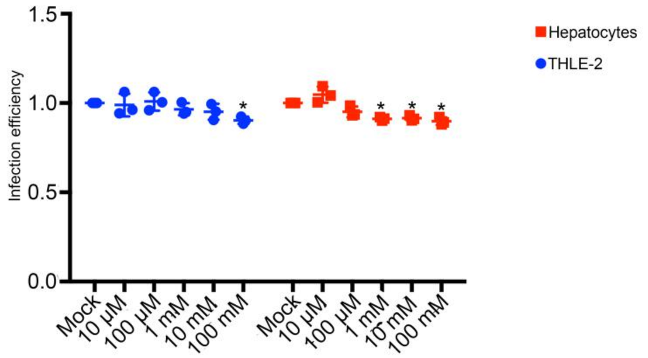

Supplementary Figure S3. N-acetyl galactosamine can prevent SARS-CoV-2 pseudovirus from THLE-2 cells and primary hepatocytes.

THLE-2 cells and primary hepatocytes were treated with 10 µM, 100 µM, 1 mM, 10 mM, or 100mM N-acetyl galactosamine and infected with SARS-CoV-2 pseudotype virus with wild-type Spike, and the infection efficiency was detected by luciferase. Each data represented the mean ± SD of three independent experiments (n=3) and were analyzed with T-test compared with mock cells. *, $p < 0.05$.

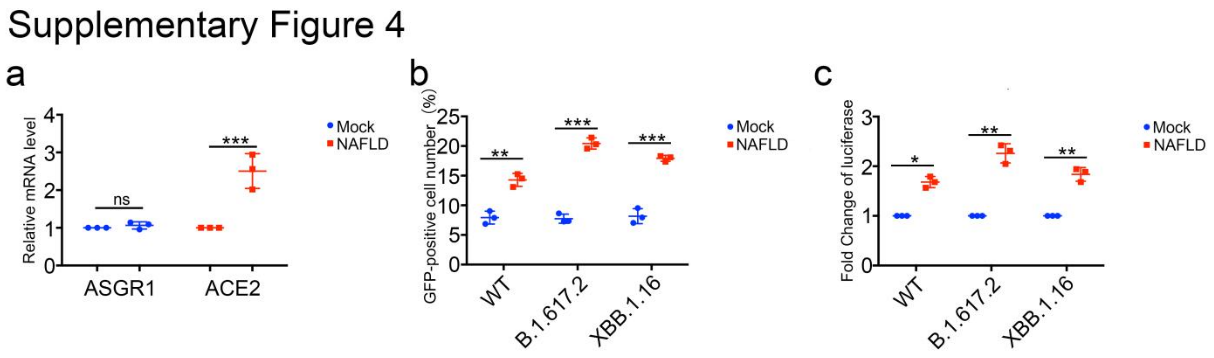

Supplementary Figure S4. SARS-CoV-2 pseudovirus infects NALFD cells.

(a) ACE2 and ASGR1 expressions were measured by qPCR. The intracellular RNA of normal or NALFD THLE-2 cells was extracted, and after reverse transcription, the corresponding primers were used to amplify ACE2, ASGR1, and GAPDH respectively. The expression levels of ACE2 or ASGR1 were finally normalized by GAPDH. **(b,c)** Normal or NALFD THEL-2 cells were infected with the GFP-labeled and luciferase SARS-CoV-2 pseudotype virus with the Spike protein of wild type, XBB.1.16 or B.1.617.2, detect the expression levels of GFP by cell flow cytometer **(b)** and luciferase by microplate **(c)** at 72 h post-infection. Each data represented the mean ± SD of three independent experiments (n=3) and were analyzed with T-test compared with mock cells. *, $p < 0.05$; **, $p < 0.01$; ***, $p < 0.001$.

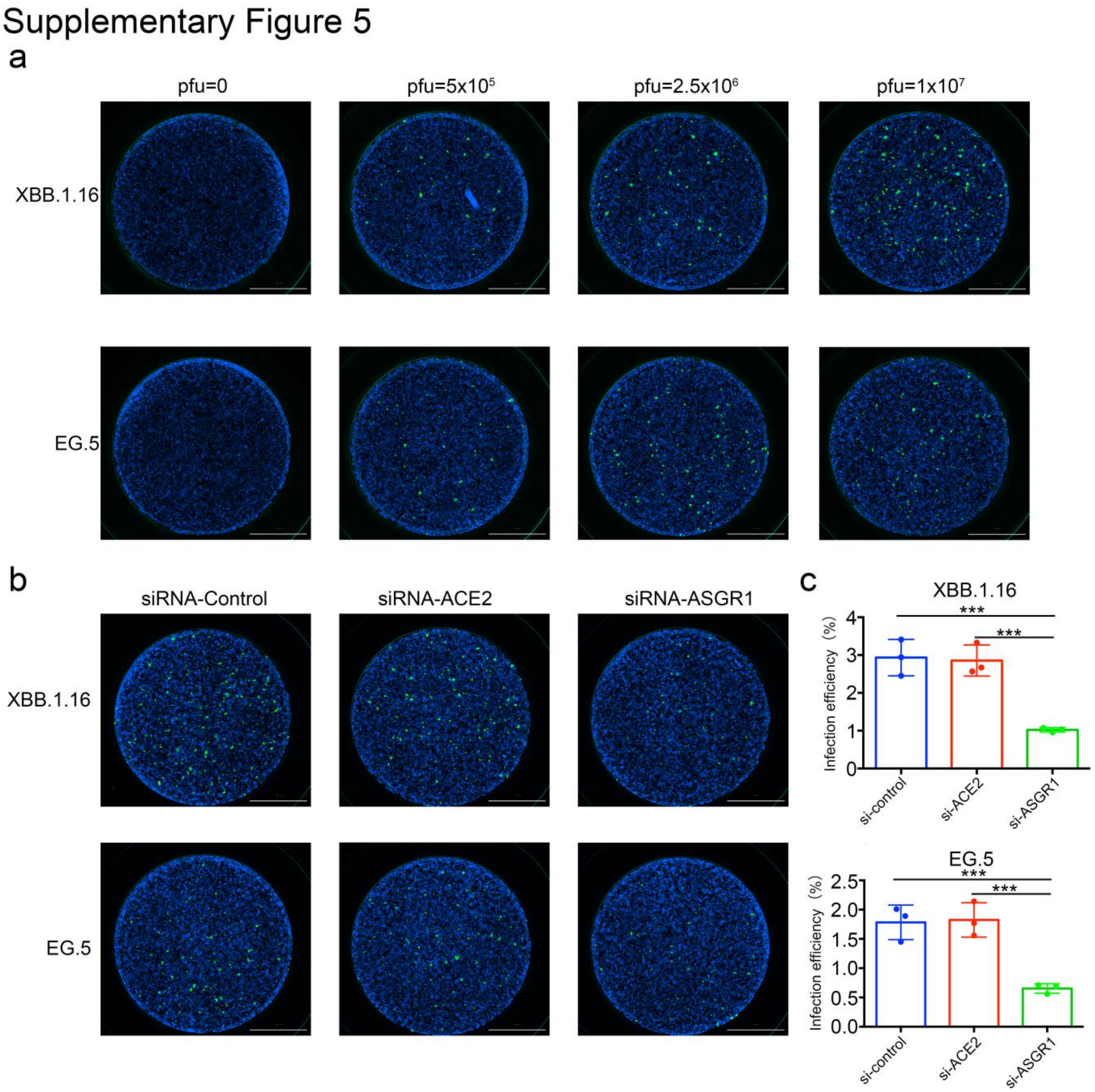

Supplementary Figure S5. SARS-CoV-2 with XBB.1.16 or EG.5 Spike infects primary hepatocytes through ASGR1. (a)

Primary hepatocytes were infected with SARS-CoV-2 with with XBB.1.16 or EG.5 Spike at different pfu (pfu=0, 5.0 x 10^5, 2.5 x 10^6, or 1 x 10^7) for 6 h and replaced the virus-free complete medium to continue culturing for 24 h. Then cells were immunostained with rabbit-anti-SARS-CoV-2 NP and DAPI. **(b, c)** siRNA targeting ASGR1 prevented SARS-CoV-2 with XBB.1.16 or EG.5 Spike from infecting liver cells. Primary hepatocytes were treated with control-siRNA, siRNA-ACE2, or siRNA-ASGR1 for 24 h, respectively. Then primary hepatocytes were infected

with SARS-CoV-2 with wild-type Spike (pfu=1 x 10^7) for 6 h and replaced the virus-free complete medium to continue culturing for 24 h. After that, the primary hepatocytes were immunostained with rabbit-anti-SARS-CoV-2 NP and DAPI. The intensity of NP were quantified using Image J and shown in **(c)**, which represents the infection efficiency of the SARS-CoV-2 with XBB.1.16 or EG.5 Spike. Each data represented the mean ± SD of three independent experiments (n=3) and were analyzed with T-test compared with cells treated with siRNA. ***, $p < 0.001$.

Supplementary Figure 6

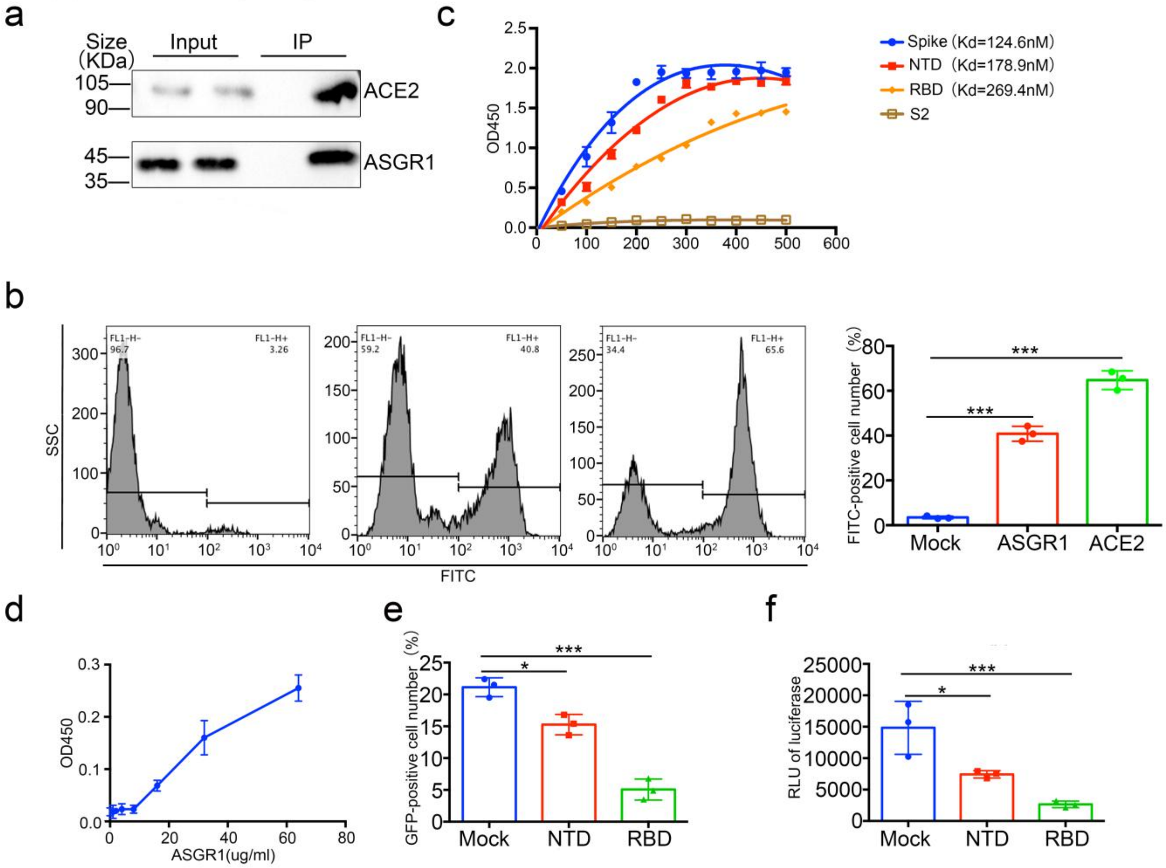

Supplementary Figure S6. ASGR1 could interact with RBD or NTD region of Spike protein.

(a) The immunoprecipitation assay was performed in 293T cell lysates transfected with HA-Spike and FLAG-ASGR1 or FLAG-ACE2 plasmids with anti-HA antibody, followed by Western blot with anti-HA or anti-FLAG antibodies. **(b)** Cell surface interaction of Spike with ASGR1 or ACE2. After co-incubation with His-Spike and 293T cells transfected with empty,

FLAG-ASGR1 or FLA-ACE2 plasmid, stained with anti-His-FITC, and analyzed by flow cytometry. The left panel is a representative flow cytometry result and the right panel is the flow statistics result. **(c)** Analysis of the effect of different regions of Spike protein on the binding of ASGR1. Different region of Spike protein was coated as an antigen in a 96-well plate, incubated with HA-ASGR1 protein with different concentration, and then anti-HA was used as the primary antibody for ELISA detection. **(d)** Competitive binding of ASGR1 to ACE2 and RBD. RBD was coated as an antigen in a 96-well plate, incubated with ACE2 protein, then incubated with HA-ASGR1 protein and anti-HA was used as the primary antibody for ELISA detection. **(e,f)** RBD protein significantly inhibits SARS-CoV-2 pseudovirus with wild-type Spike infection. 293T-ASGR1 cells were treated with NTD or RBD in advancd, then infected infected with the GFP-labeled and luciferase SARS-CoV-2 virus pseudotype with wild-type Spike, detected the expression levels of GFP by cell flow cytometer **(e)**, luciferase by microplate **(f)** at 72 h post-infection. Each data represented the mean ± SD of three independent experiments (n=3) and were analyzed with T-test compared with mock cells. *, $p < 0.05$; ***, $p < 0.001$.

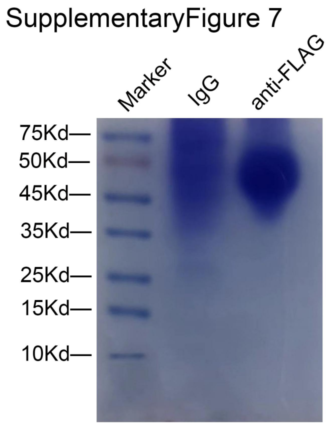

Supplementary Figure S7. The FLAG-ASGR1 protein was obtained from the 293T cell lysate transfected with FLAG-ASGR1 by co-immunoprecipitation, and the purified Flag-ASGR1 protein was identified by SDS-PAGE and Coomassie brilliant blue staining.

Supplementary Figure 8

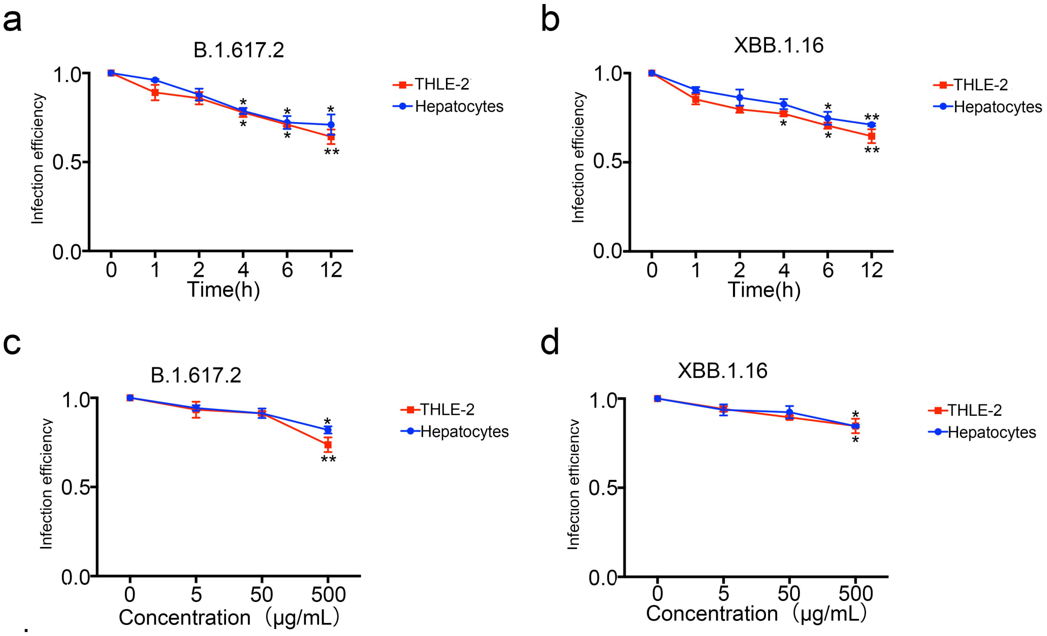

Supplementary Figure S8. Soluble ASGR1 protein can prevent SARS-CoV-2 pseudovirus from infecting liver cells.

(a,b) The SARS-CoV-2 pseudotype virus with B.1.617.2 or XBB.1.16 Spike was incubated with 500 μg/mL soluble ASGR1 for 0 h, 1 h, 2 h, 4 h, 8 h, and 12 h **(a)** or with 0 μg/mL 5 μg/mL, 50 μg/mL and 500 μg/mL for 6 h **(b)**, then infected THLE-2 cells and primary hepatocytes. The infection efficiency was detected by luciferase. Each data represented the mean ± SD of three independent experiments (n=3) and were analyzed with T-test compared with 0 h or 0 μg/mL treated with cells. *, $p < 0.05$; **, $p < 0.01$.

Supplementary Figure 9

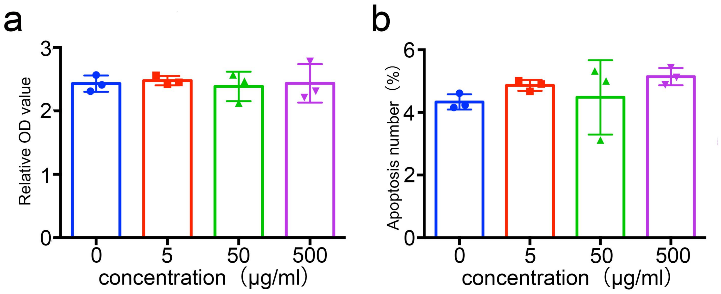

Supplementary Figure S9. Effect of soluble ASGR1 protein on the proliferation and apoptosis of lung epithelial cells.

(a) Cell proliferation of BEAS-2B cells incubated with 0 μg/mL, 5 μg/mL, 50 μg/mL, and 500 μg/mL soluble ASGR1 protein for 24 h. Cells were analyzed by CCK8. **(b)** Apoptosis of BEAS-2B cells incubated or not with 500 μg/mL soluble ASGR1 protein for 24 h was measured by TUNEL staining, followed by flow cytometry. Each data represented the mean ± SD of three independent experiments (n=3) and were analyzed with T-test compared with 0 μg/mL treated with cells.

Supplementary Figure 10

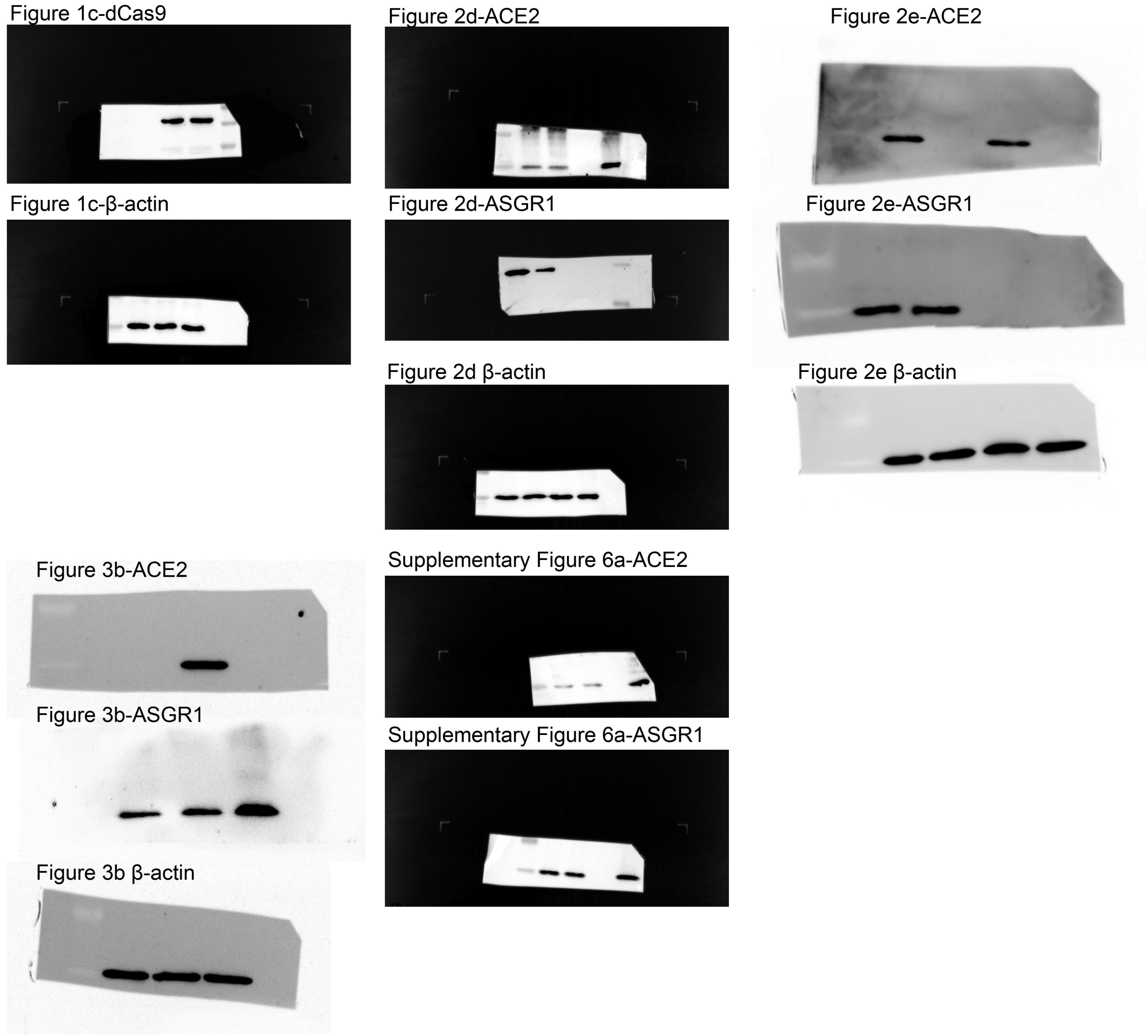

Supplementary Figure S10. Full uncut original pictures.
